# What influences food choices in anorexia nervosa? Disentangling cognitive and emotional components of decision-making by translational research

**DOI:** 10.1016/j.nsa.2024.104080

**Published:** 2024-06-22

**Authors:** Laura Di Lodovico, Héloise Hamelin, Lucas DeZorzi, Chloe Tezenas du Montcel, Erik Schéle, Iris Stoltenborg, Roger Adan, Suzanne Dickson, Philip Gorwood, Virginie Tolle, Odile Viltart

**Affiliations:** a Clinique des Maladies Mentales et de l’Encéphale, GHU Paris Psychiatrie et Neurosciences, Hôpital Sainte-Anne, F-75014, Paris, France; b Université de Bordeaux, INSERM, Neurocentre Magendie, U1215, F-33000, Bordeaux, France; c Université de Paris, INSERM, Institut de Psychiatrie et Neuroscience de Paris (IPNP), U1266, F-75014, Paris, France; d McGill University, Douglas Institut universitaire en santé mentale, Département de Psychiatrie, Montréal, QC, H4H 1R3, Canada; e Université de Lille, CNRS, UMR 9193, SCALab - Sciences Cognitives et Sciences Affectives, PsySEF Departement, U9193, F-59653, Villeneuve d’Ascq, France; f Institute of Neuroscience and Physiology, The Sahlgrenska Academy at the University of Gothenburg, Medicinaregatan 11, SE-405 30, Gothenburg, Sweden; g Department of Translational Neurosciences, University Medical Center Utrecht, Universiteitsweg 100, 3584, CG, Utrecht, the Netherlands

**Keywords:** Anorexia nervosa, Anxiety, Food choices, Decision-making, Reward, Animal models

## Abstract

Anorexia nervosa is a serious mental illness characterized by voluntary restriction of food intake and avoidance of high-calorie food. Anxiety, highly comorbid with anorexia nervosa, appears to be a significant, yet underexplored, factor affecting core behavioural symptoms such as food restriction and compulsive physical exercise. The aims of this review are to disentangle the influence of anxiety in food decision-making in anorexia nervosa and to offer a comprehensive model connecting the mechanisms involved.

The shift from food approach to avoidance seems to be a conditioned response, underpinned by an activation of salience and fear circuitry. Altered neurotransmission (dopamine, serotonin) and neuroendocrine release (leptin, ghrelin, cortisol), aberrant neural structure activation (hyperactivation of the amygdala and hypoactivation of the insula-ventral striatum circuit) and cognitive and behavioural traits shared by anxiety and anorexia nervosa (rigidity, compulsiveness) contribute to these modifications. Animal models suggest a bidirectional relationship between food restriction and anorectic-like behaviours, strengthening yet complexifying the link between anxiety and food choice in anorexia nervosa. Therapeutic strategies focusing on anxiety and the conditioned response could contribute to restore healthy food choices and dissociate food stimuli from the anxious response elicited.

## Highlights

• Neuroendocrine and cognitive factors affect decision-making in anorexia nervosa.
• Anxiety contributes to altered food decision-making in anorexia nervosa.
• Altered salience network triggers a switch from desire to dread for food.
• Metabolic neuropeptides influence anxiety and food decision in anorexia nervosa.
• Cognitive-behavioural therapies may restore healthy food choice in anorexia nervosa.

## Abbreviations

ABA =: activity-based anorexia
ABMT =: attention bias modification training
AgRP =: Agouti-related protein
BLA =: basolateral amygdala
BMI =: body mass index
CeA =: central nucleus of the amygdala
CRH =: corticotropin releasing hormone
EPM =: elevated plus maze
HPA =: hypothalamus pituitary adrenal axis
LHA =: lateral hypothalamic area
LepR =: leptin receptor
MGT: Mouse Gambling Task
NAcc =: Nucleus Accumbens
NPY =: neuropeptide Y
OCD =: obsessive compulsive disorder
OFC =: orbitofrontal cortex
PFC =: prefrontal cortex
VTA =: ventral tegmental area
5-HT =: 5-hydroxytryptamine or serotonin

## 1 Introduction

Anorexia nervosa is a psychiatric disorder characterised by a pervasive fear of gaining weight or becoming obese, underpinned by a distorted body image and an overvaluation of weight and shape. Behavioural efforts to control weight and shape are at the core of the pathology and involve voluntary restriction of food intake. In most cases, patients also engage in compulsive physical exercise and resort to purging behaviours such as vomiting or misuse of laxatives. The ensemble of weight and shape-controlling behaviours leads to an underweight status, which is often under-recognised or denied (American Psychiatric Association, 2013; Treasure et al., 2020).

Five-year recovery rates for anorexia nervosa are estimated to be under 70%, and remission is reported in 19–65% of patients (Smink et al., 2013). Up to 50% of patients encounter relapse (Khalsa et al., 2017) and lethality is among the highest for psychiatric diseases, with crude mortality rates of 5.1 deaths per 1000 person-years (Arcelus et al., 2011), mainly accounted for by somatic complications and suicide (Kask et al., 2016).

Refeeding and normalisation of eating behaviour are the cornerstones of treatment. Notwithstanding, the management of anorexia nervosa is challenging and time-consuming. While some progress has been made in the treatment of children and adolescents by family-based approaches (Byrne et al., 2017; Le Grange and Heisler, 2009), evidence is still too scarce to determine the superiority of a given treatment approach in the management of adults (Hay et al., 2019; Zipfel et al., 2015). Multidisciplinary approaches are the rule, based on the coordination of different healthcare disciplines, such as internal physicians, psychiatrists, psychologists and nutritionists (Monteleone et al., 2023). Psychiatric comorbidities are an important factor of treatment failure, increased severity and higher mortality (Kask et al., 2016; Li et al., 2023).

Depression and anxiety are the most frequent comorbidities of anorexia nervosa (Godart et al., 2002). Anxious manifestations are present in almost 75% of patients with anorexia nervosa, in the form of anxious symptoms, trait anxiety and anxiety disorders (Godart et al., 2002; Lloyd et al., 2018). Anxiety has been already identified as a potential etiological factor of anorexia nervosa, and as a vulnerability trait that often persists after remission (i.e., associated to anorexia nervosa as a trait) (Kaye et al., 2004; Radix et al., 2023). Shared transmission between anxiety and eating disorders is largely supported in the current literature (Keel et al., 2005). Behavioural symptoms of anxiety may be so highly entrenched with those of anorexia nervosa that it may be challenging to identify whether anxiety serves as a primer for anorexic behaviours or the other way round. Analogously, it is sometimes challenging to understand whether core symptoms of eating pathology underpin or alleviate (acquiring the forms of “safety behaviours”) the recurring fears experienced by patients, such as gaining weight or becoming fat by eating. Examples of safety behaviours (i.e., actions underpinned by fear and that reduce or prevent negative feelings such as anxiety) are food restriction and avoidance, that become maladaptive to the extent of that they expose the patient to short- and long-term disabling consequences (Spix et al., 2023). The implementation of repetitive exercise regimes can also act as a safety behaviour, filtering out potentially destabilising affects and reducing levels of arousal (Holtkamp et al., 2004).

Compulsive exercise plays a significant part in the perpetuation of anorexia nervosa, with the anxiolytic properties of the exercise practice contributing to this phenomenon (Meyer et al., 2011). The engagement in compulsive exercise despite malnutrition is underpinned by mechanisms of positive and negative reinforcement. These include an increase in positive emotions induced by exercise (Di Lodovico et al., 2021), and the avoidance of anxiety in the absence of alternative and healthier coping strategies (Lavender et al., 2015). It is pertinent to highlight that levels of compulsive exercise are predicted by food restriction and anxiety, thereby confirming its pivotal role in the pathophysiology of anorexia nervosa and its function as a maladaptive coping strategy against anxiety (Holtkamp et al., 2004).

The centrality of ritualistic behaviours (i.e., eating behaviours, exercise routines) and obsessional preoccupations about weight and shape inspired associations between anorexia nervosa and obsessive compulsive disorder (OCD) (Halmi et al., 2003). It is well established that OCD is highly comorbid with anorexia nervosa. It is estimated that approximately 40% of patients with anorexia nervosa also meet diagnostic criteria for OCD (Halmi et al., 2003; Levinson et al., 2019; LaSalle et al., 2004), and that 68% of patients with the restricting type of anorexia nervosa and 79% of patients with the binge eating-purging subtype had at least one obsession or compulsion in their lives (Halmi et al., 2003). The genetic association between the two disorders is strong (∼45%) (Watson et al., 2019). Some authors have proposed that safety behaviours may be considered a form of compulsion. Furthermore, the majority of symptoms associated with anorexia nervosa, including exercise, body checking, and restriction, appear to exhibit a compulsive nature (Levinson et al., 2019; Coniglio et al., 2017). This co-occurrence of compulsive-like behaviours and vulnerability to self-starvation and weight loss has also been found in mice tested in the Activity Based Anorexia (ABA) model (Favier et al., 2020). Obsessions have been identified as strong predictors of the drive for thinness and are reported as the most disturbing symptom by patients with anorexia nervosa, suggesting the importance of addressing obsessions to relieve patients from anxiety (Levinson et al., 2019). The relationship between OCD and anorexia nervosa is complex due to the potential for bidirectional influences. Obsessive-compulsive behaviours related to food are found to be particularly prevalent in individuals experiencing starvation and prolonged food restriction (Pallister and Waller, 2008).

Restriction of food intake is a central symptom of anorexia nervosa, predominantly responsible for the clinical severity of the disease. Food restriction consists in a progressive reduction of the amount of food intake, meal skipping and eventually aphagia in the most severe cases (quantitative food restriction). In these patients, food choice is biased towards low-calorie foods, while high-calorie and high-fat/sugar foods are avoided (qualitative food restriction) (Treasure et al., 2020; Zipfel et al., 2015). The relentless pursuit of food restriction along the protracted course of the disease leads to the aggravation of weight loss. Compulsive exercise exacerbates this weight loss. This usually results in a pathological escalation of exercise in parallel with poor weight intakes (Dalle Grave et al., 2008). Several theories have been suggested to explain this counterintuitive and persistent alteration of food choice in anorexia nervosa, focusing on the neuroendocrine dysregulation of satiety and feeding (Viltart et al., 2018), on cognitive abnormalities such as an alteration of the reward system (Frank, 2021), on habit formation, accounting for the rigidity and persistence of eating choices (Compan et al., 2015) and aberrant processes of decision-making (Guillaume et al., 2015; Di Lodovico et al., 2022).

Several publications have stressed the existence of maladaptive processes of decision-making in anorexia nervosa (Guillaume et al., 2015; Di Lodovico et al., 2022; Na et al., 2019; Verharen et al., 2019) and provide a framework according to which maladaptive food choices could be read as the result of the preference of short-term relief from anxiety over long-term aggravation of malnutrition (Di Lodovico et al., 2022). Emotion is identified as one of the main factors influencing decision-making (Bechara, 2000; BecharaAntoine and Damasio, 2005). Moreover, severe trait anxiety has been found to impair decision-making performance (Zhang et al., 2015) and to shift reliance from a goal-directed attentional system to one that is stimulus-driven (Zhang et al., 2015; Corbetta and Shulman, 2002). One study found that the impairment in decision making reported by patients with anorexia nervosa was underpinned by high levels of anxiety (Fornasari et al., 2014). Accumulating evidence, therefore, suggests a complex but significant interplay between anxiety, eating psychopathology and altered decision-making capacity at the origin of maladaptive food choice in anorexia nervosa. Despite the strength of this evidence, no comprehensive theoretical model has been proposed yet to organize the different findings on the relationship between anxiety and food decision-making in anorexia nervosa.

This review aims to summarise the clinical and scientific evidence exploring the role of anxiety on food decision-making in anorexia nervosa. We will dissect the process of decision-making and its features, to provide a comprehensive model of the effect of anxiety on food decision-making. Finally, we will integrate this model with the others existing in the current research landscape of anorexia nervosa.

## 2 A role for anxiety in food decision-making in anorexia nervosa

### 2.1 Anxiety and food restriction: evidence from clinical research

When healthy individuals are presented with food, they perceive it as a reward, especially when hungry (Kaye et al., 2020). However, in individuals applying dietary restraint, food preference depends less on the pure hedonic responses to taste; cognitive mechanisms of control and regulation of autonomic functions, emotions and cognitions are powerful enough to override homeostatic needs in situations of food exposure and choice (Stoner et al., 1996; Young et al., 2020).

Food choice in anorexia nervosa displays some recurring features such as a preference for low-calorie over high-calorie food and lower desire to eat high-calorie and high-fat food. The latter is avoided (Treasure et al., 2020; Di Lodovico et al., 2023), rated as less healthy and less tasty, less wanted, less liked (Lloyd and Steinglass, 2018) and rarely chosen in food-choice paradigms (Stoner et al., 1996). Size and caloric content of food are overestimated and rated as intolerable and anxiogenic (Lloyd and Steinglass, 2018).

Thus, it would appear that food avoidance in anorexia nervosa is not underpinned by a decrease in the hedonic value of food (liking) but rather, by aberrant motivation (i.e., wanting) towards low-calorie food (Cowdrey et al., 2013) and by a reluctance to eat particular categories of food by virtue of the beliefs concerning them (Stoner et al., 1996; Uniacke et al., 2020). These beliefs are powerful enough to drive food choice in ways that over-ride homeostatic and reward processing.

It is highly plausible that anxiety and the fear of being fat underpin food avoidance; individuals with anorexia nervosa are highly anxious in general (Marzola et al., 2017) and notably, the primary focus of this anxiety is food-related (Forrest et al., 2018).

Given the anxious response elicited by food-related stimuli, modern learning theories suggest that food choices are a manifestation of conditioned avoidance in anorexia nervosa. In other words, food avoidance may be understood as an acquired, learned response developed from vulnerabilities in emotional learning and memory processing (Garcia-Burgos et al., 2023). Two main associative processes could account for food avoidance: conditioned fear (e.g., of catastrophic weight gain) and conditioned flavour aversions (e.g., being potentially associated with gastrointestinal discomfort) (Garcia-Burgos et al., 2023). With the repetition of food avoidance, anxiety itself may become the trigger of restriction by a stimulus-response association, that is typical of compulsive and habit-driven behaviours (Krypotos et al., 2015; Lloyd et al., 2017).

Food avoidance has a central role in the morbidity and mortality of anorexia nervosa (Uniacke et al., 2020; Schebendach et al., 2011). This behavioural disturbance leads to severe restrictions of intake, with extensive impact on the body and the brain. Hence, the priority is to focus on this symptom by appropriate treatment. Eating-related anxiety has been proposed as a target (Steinglass et al., 2011), but the underpinning mechanisms have not been delineated (Murray et al., 2016). The question of what supports the broad and severe food restriction in anorexia nervosa is still unsolved.

Alterations in dopamine reward signalling have been suggested to play a pivotal role in the disruption of the balance between approach and avoidance towards food. Functional magnetic resonance imaging studies report higher dopaminergic activity in the food intake-control circuitry, strengthening the connectivity between ventral striatum and hypothalamus, and this may explain the ability of patients to override hunger cues (Frank, 2021). Anxious conditioning to food intake may trigger the recruitment of dopaminergic circuits to engage in fearful avoidance as opposed to food approach (Young et al., 2020). Moreover, dietary restriction has an impact on serotonin concentrations by a reduction of the intake of its precursors (i.e., tryptophan) (Gauthier et al., 2014), supported by an increase of 5-HT1a receptors availability in parallel with the decrease of its endogenous ligand (Galusca et al., 2008). Serotoninergic dysfunctions may, however, have other causes than exogenous depletion of its precursors by starving. Decreased synaptic release, abnormal receptor regulation and the abnormal density of 5-HT receptors could also precede the development of anorexia nervosa, giving rise to the hypothesis of a 5-HT related organic background in this pathology (Galusca et al., 2008).

In patients with anorexia nervosa, passive exposure to the visual food stimuli activates the attentional salience network that encompasses the amygdala, insula and anterior cingulate cortex (Young et al., 2020; Joos et al., 2011). While healthy subjects only reacted to calorie-dense food images, patients with anorexia nervosa showed a generalized attentional bias for food, that is even more intense when food is rich in calories (Mercado et al., 2020), potentially explaining the persistence of food-related concerns (Blechert et al., 2011).

According to eye-tracking data, while initial orientation to food cues is comparable to healthy controls, patients tend to avoid maintaining attention on food cues at later stages of attentional processes. This attentional avoidance of food is more pronounced in adults with longer illness duration, suggesting this behaviour as a maintaining factor of the disorder (Werthmann et al., 2019). The startle response, a marker of sensitivity of appetitive responses to (food) stimuli, was not attenuated in patients presented with food stimuli. This suggests reduced sensitivity of the appetitive system to food cues and inability to experience pleasure from food (Friederich et al., 2006). These behavioural features (i.e., vigilance, avoidance, low appetitive sensitivity), and their neurobiological underpinnings (in particular, the activation of the attentional salience network, common to anxiety and fear) (Young et al., 2020; Gizewski et al., 2010), may account for the switch from “desire” to “dread” elicited by food in anorexia nervosa (Frank, 2021). Finally, in response to sucrose expectation, patients with anorexia nervosa show a strong vigilance response, with a hyperactivation of the amygdala and the reward circuit. Trait anxiety inversely modulated this correlation, so that in case of anxiety the activation of the reward circuit is dampened (Frank et al., 2023).

In summary, the presentation of food cues may elicit dread and negative emotions instead of the appetitive response orchestrated by ventral striatal activation in healthy subjects. While eating would stimulate mood dysphoria, dietary restraint and reduced daily caloric intake show anxiety-reducing properties in patients with anorexia nervosa (Steinglass et al., 2010). To cope with anxiety, patients revert to “safety behaviours”, amongst which the most relevant to food choice are: eating particular food combinations, separating foods, preparing their own meals and leaving food on the plate (Simonazzi et al., 2023). This defensive response is orchestrated by the activation of the amygdala and the prefrontal-striatal interplay, consolidating the hyperactivation of dorsolateral striatum and thus, habit formation. This may explain, in turn, the rigidity of food choices, safety behaviours and food-related rituals in patients. With time and illness progression, these behaviours become habit-driven, highly entrenched mechanisms in the response to food-related stressors. Putting stress at the core of eating disorder onset, the hypothesis is that food restriction would represent a maladaptive decision to cope with difficult-to-manage stressors (Walsh, 2013). Food restriction thereby confers the impression of self-control, at least temporarily relieving patients from anxiety.

### 2.2 Anxiety and food restriction: inputs from fundamental research

Anxiety, defined as a future-oriented state primed by distal and potential threat, is characterised by neurobehavioral responses such as arousal and vigilance (Daviu et al., 2019; Murray et al., 2018). Chronic and/or acute stressors, from early life onwards, are among the main triggers of anxiety and represent a vulnerability factor for anorexia nervosa (Pike et al., 2008). The relationship between anxiety and disruption of feeding choices has been widely explored in animal models. In particular, the “Activity Based Anorexia” (ABA) model, developed by Routtenberg and Kuznezof in the 1960s, combines activity in a running wheel and time-restricted food access (e.g. 1–3 h per day) on isolated rodents, leading quickly to self-starvation and increasing maladaptive physical activity (Adan et al., 2011; Routtenberg and Kuznesof, 1967; Méquinion et al., 2015a; Epling et al., 1983). Because this model mimics two main aspects of anorexia nervosa (i.e., food restriction and physical hyperactivity), it is still considered as a gold standard. Behavioural approaches assessed anxiety levels in mice submitted to the original ABA protocol by the elevated plus maze or the open field. In the original ABA protocol, rodents did not survive more than 5–6 days, making it impossible to study the effects of the combination of food restriction and free running activity in the long term. This led to the development of several modified ABA protocols, to mimic more precisely physiological aspects of the chronic starvation observed in anorexia nervosa (Méquinion et al., 2015b; Schalla and Stengel, 2019). Recently, Schwenzer et al. (2022) modified the initial ABA protocol by reducing the amount of food delivered (equivalent to 30% of the *ad libitum* food intake during the habituation period) instead of reducing the feeding time. They showed that rats displaying higher levels of anxiety before food restriction displayed increased vulnerability to hyperactivity and weight loss, while rats undergoing food restriction showed a decrease of anxiety-like behaviours after undergoing the ABA paradigm (Schwenzer et al., 2022). Thus, food restriction is known to reduce anxiety, as measured in the elevated plus maze, and physical hyperactivity is associated with increased anxiety (Schwenzer et al., 2022; Wable et al., 2015) in the ABA model as in patients (Holtkamp et al., 2004; Sternheim et al., 2015). It would appear that negative energy balance unmasks the relation between increased anxiety and hyperactivity. Similar results have been described in another ABA modified paradigm where mice, housed by cages of two, fed with 50% of their daily intake and with free access to a running wheel, displayed all the metabolic changes observed in patients with anorexia nervosa (Méquinion et al., 2015a; Duriez et al., 2020a). At the end of the two weeks of protocol, despite no straightforward anxiety-like phenotype, physical activity associated with chronic food restriction improved coping strategies in an anxiogenic environment (Duriez et al., 2020b). The relationship between proxies of behavioural symptoms of anorexia nervosa (food restriction, physical hyperactivity) and anxiety-like behaviours seems thus bidirectional. As in humans (Kaye et al., 2004), anxiety can precede the onset of behaviours typical of anorexia nervosa.

Despite its value to assess the neurobiological and neuroendocrine mechanisms of anorexia nervosa development and maintenance (Schalla and Stengel, 2019), the ABA model *per se* does not allow us to explore the role of anxiety in food choice, since food restriction is imposed and controlled by the experimenter (Hurel et al., 2019). An additional approach to assess the effects of stress and anxiety on feeding, is to focus on the physiological endocrine responses to stress, such as hypercortisolemia induced by the activation of the hypothalamo-pituitary-adrenal (HPA) axis. The artificial increase of plasma concentrations of glucocorticoids, like cortisol (in human)/corticosterone (in rodents), as well as its fluctuations in response to a stress, were found to induce changes in food choice, even at a qualitative level (Cabeza et al., 2021). Glucocorticoids are key factors in the mobilisation of energy resources during fasting, caloric restriction and/or prolonged exercise; they stimulate lipolysis and increase protein catabolism when in negative energy balance. This explains the unsurprising surge of plasma corticosterone concentrations and the long-lasting increases in HPA axis activity, no matter which ABA protocol is used (Méquinion et al., 2015a; Duriez et al., 2020b; Duclos et al., 2013; Burden et al., 1993; Kinzig and Hargrave, 2010). Both in patients and in rodent models, physical hyperactivity and/or excessive exercise in conditions of food restriction might participate in the central activation of the stress response through hypothalamic corticotropin-releasing hormone (CRH) neurons. This neurohormone plays a pivotal role in the onset and development of the neurological, behavioural, endocrine and immunological response to stress (Diane et al., 2008; Dunn and Berridge, 1990) by inhibiting feeding in stressful situations such as acute or chronic food deprivation, likely by potentiating the anxiogenic effect (Dunn and Berridge, 1990; Sekino et al., 2004). Acute stress is thus considered to suppress food intake and body weight gain (Duclos et al., 2013; Diane et al., 2008; Berner et al., 2019). The link between glucocorticoids, physical activity and feeding is bidirectional: food restriction and/or physical activity promote a surge in plasma corticosterone levels, and corticosterone is believed to play a role in macronutrient selection. For example, it was shown that larger glucocorticoid doses promote fat consumption, while relatively lower doses increase carbohydrate consumption (Bligh et al., 1993). The effects of glucocorticoids and/or stress on food selection are also mediated by factors such as insulin concentration (la Fleur, 2006) and stress sensitivity (Diane et al., 2008; Teegarden and Bale, 2008). Furthermore, acute stress (15 min of forced swimming on three consecutive days) involved an immediate but transitory change in food intake quantity and composition. Three hours after an acute stress, energy intake was significantly decreased. Interestingly, female rats had a specific decrease in fat intake, showing that stress is not only responsible for a decrease of energy intake but also for a change in the composition of macronutrient intake (Diane et al., 2008).

Similar to what is observed in animal models, patients display high plasma cortisol concentration and a higher cortisol responses 30 min after awakening (Monteleone et al., 2016, 2017). These two phenomena, interpreted as an adaptive response to the chronic stress caused by energy deprivation, appear to regress by weight normalisation. More specially, in patients with acute anorexia nervosa, high cortisol levels were correlated with decreased hunger and desire to eat hedonic food. This raises the hypothesis that HPA dysfunction, and in particular altered concentrations of the anorexigenic neurohormone CRH, may reduce the appetitive and hedonic drive towards food in patients with anorexia nervosa (Lawson et al., 2013). Chronic hypercortisolemia is thus an adaptation to the metabolic stress caused by underfeeding, but also a likely response to other sources of stress. The persistence of HPA axis dysregulation after remission may be secondary to the psychological burden provided by eating psychopathology and comorbidities (Schmalbach et al., 2020), in addition to the metabolic stress caused by transient drops in blood glucose.

## 3 Food decision making in anorexia nervosa: features and factors involved

### 3.1 What is decision making?

Decision making is essential for survival in everyday life (Enax and Weber, 2016). Choices are mostly characterized by uncertainty and take into account physiological needs and the contextual environment. According to Doya et al., (Duclos et al., 2013), decision-making can be decomposed in four steps: (1) perception and recognition of the choice situation, (2) cost and benefit evaluation of each option (i.e.: expected value of the outcome), (3) choice selection and action, and (4) evaluation of the real value of the outcome. The comparison between the real and the expected value of the outcomes allows learning of the choice-outcome association and improving future decisions in similar contexts. The cerebral structures specifically involved in each decisional step are the orbitofrontal cortex (OFC), the amygdala, the dorsal and ventral striatum, and the insular and cingulate cortex (Watson et al., 2019; Burden et al., 1993; Kinzig and Hargrave, 2010).

Food decision making involves the valuation of the available food items, and then the selection of the most valued (Zald, 2009). In humans, the valuation of food items often occurs well before the food items are available, which suggests that it is also based on the internal representations of food items and the expected sensory experiences associated. The valuation of the expected food reward is mostly accounted for by the OFC, and integrated with other interoceptive and external variables, such as other available food items, the context, whether this food item has been eaten before and the internal state of satiety (Zald, 2009; Tremblay and Schultz, 1999). Consequently, diminished interoceptive awareness in anorexia nervosa (Khalsa et al., 2018) contributes to less performant decision-making. Patients display a less performant moment-by-moment mapping of the body's internal landscape across conscious and unconscious levels, impacting on the stability of internal states and motivated behaviour. Impaired sensing of a depleted nutritional state may contribute, by this means, to guide a less performant food-seeking behaviour and consumption (Maniscalco and Rinaman, 2018).

The disruption of the interplay between the different components of food decision likely occurs at different sites, starting from the physiological factors involved in the neuroendocrine signalling in the hypothalamus. Anorexia nervosa is reconceptualized as a “metabo-psychiatric disorder”, stressing the role of the alteration of core metabolic systems concurring to the clinical manifestation of the disease (Bulik et al., 2021). Indeed, physiological factors, such as circulating hormones (i.e. leptin, ghrelin) inform the nervous system of its actual nutritional status, and regulate the balance between hunger and satiety. In particular, two hypothalamic pillars (Agouti Related Protein, AgRP, and orexin neurones) are identified in the neural control of feeding and energetic balance.

### 3.2 Metabolic components

#### 3.2.1 Agouti-related protein

Anorexia nervosa is associated with failure to recognize hunger and nutritional needs. An increasing number of studies suggest that these behavioural symptoms likely involve AgRP neurons. AgRP neurons, located in the hypothalamic arcuate nucleus, signal energy deficit to promote hunger. Acute activation of AgRP neurons induces feeding while their specific ablation or silencing results in profound anorexia (Luquet et al., 2005; Krashes et al., 2011). Besides promoting food consumption, activation of AgRP neurons elicits learned instrumental food-seeking, indicating that these neurons are an entry point to motivational processes resulting from homeostatic deficit (Sternson et al., 2013). When food is not available, optogenetic stimulation of AgRP neurons creates a state of aversion, suggesting that AgRP neurons, besides increasing food intake, also encode the negative valence (i.e. unpleasant feelings) associated with hunger (Betley et al., 2015). Furthermore, AgRP neurons modulate mesolimbic dopamine activity and food palatability (Luquet et al., 2005; Krashes et al., 2011), related to the motivational processes at the origin of food seeking. The decision to engage in food-seeking behaviour is influenced not only by homeostatic signals related to energy deficits, but also by the presence of competing motivational drives and learned cues signalling food availability. Stimulation of AgRP neurons is therefore sufficient to drive food-seeking behaviour in the presence of a competing motivational drive, such as threat avoidance. However, the decision to engage in certain behaviours and not others (e.g. food seeking versus avoidance) in response to AgRP stimulation depends on the temporal priority of one motivational drive relative to the onset of a competing drive (Jikomes et al., 2016). Choice behaviour is also characterised by temporal discounting, i.e. a preference for immediate rewards when faced with a choice between immediate and delayed rewards. Energy balance status modulates temporal discounting, as hungry mice strongly prefer immediate food rewards, whereas satiated mice are indifferent to reward delay (Li et al., 2021). It is noteworthy that AgRP neurons have been shown to participate in the decision-making process. The optogenetic activation of AgRP neurons or their axon terminals within the bed nucleus of stria terminalis (a part of the extended amygdala involved in the regulation of stress, anxiety and feeding) induced temporal discounting in sated mice. Furthermore, this activation, which mimicked the effect of a fasting state, resulted in a bias towards impulsive decision-making (Li et al., 2021). These data suggest a pivotal role for the hypothalamus in influencing choice behaviour and food decision making via AgRP neurons signalling. Indeed, situations mimicking negative energy balance, such as chronic food restriction or acute chemogenetic activation of AgRP neurons, can also influence social decision-making, by attenuating pro-social behaviours (Pozo et al., 2023). Other behavioural phenotypes of anorexia nervosa are likely to be influenced by the activity of AgRP neurons. For instance, in the absence of food, activation of AgRP neurons in mice alters behavioural flexibility as measured by a modified Barnes maze test (Zimmer et al., 2019), and elicits repetitive and compulsive behaviour (Dietrich et al., 2015; Miletta and Horvath, 2023; Miletta et al., 2020). Interestingly, stereotypic behaviours induced by AgRP neurons are also associated with decreased anxiety. In humans, data involving AgRP in symptoms of anorexia nervosa are still sparse. Variants in the AgRP gene were found to be associated with anorexia nervosa and low BMI (Vink et al., 2001; Dardennes et al., 2007; Yilmaz et al., 2014). In another study, reward association learning, studied as a measure of cognitive flexibility in adolescent patients with anorexia nervosa, was correlated with abnormal circulating levels of AgRP (Sarrar et al., 2011). Although these data suggest that AgRP neurons may be a key neuronal target for therapeutic interventions related to symptoms of anorexia nervosa, further explorations are needed. Interest in AgRP neurons is also linked to the fact that they are targeted by peripheral metabolic signals such as ghrelin or leptin, both of which are implicated in motivational and decision-making processes.

#### 3.2.2 Leptin

Leptin is a hormone produced by white adipose tissue, controlling satiety and regulating fat storage. In anorexia nervosa, levels of leptin are extremely low (Karageorgiou et al., 2020). Low circulating levels of leptin, leading to increased levels of neuropeptide Y (NPY), result in the pathological hyperactivation of the HPA axis (Zhang et al.). Mice that lack leptin (*ob/ob* mice), despite an enormous drive to eat, display food neophobia (Finger et al., 2010). Leptin receptors (LepR) are expressed in a subpopulation of ventral tegmental area (VTA) dopamine neurons (Omrani et al., 2021; Leshan et al., 2010) that project mostly to central nucleus of the amygdala (CeA). These neurons play a role in anxiety. Leptin is known to reduce anxiety (Liu et al., 2010). Removal of LepR signaling (by removing Stat3) from dopamine neurons results in hyperactivity in male mice (Fernandes et al., 2015) and leads to anxiety in females (Fernandes et al., 2021). This gender difference is relevant as anorexia nervosa occurs more frequently in women. In female mice lacking LepR signaling on dopamine neurons (not exposed to ABA), a D1 antagonist in the CeA normalized the increased anxiety (Fernandes et al., 2021). This highlights the importance of dopamine in different regions of the amygdala (CeA *versus* basolateral amygdala, BLA) in the control of anxiety (de la Mora et al., 2010), as recent findings showed that increasing dopamine signaling in BLA has anxiolytic effects (Nguyen et al., 2021; Morel et al., 2022). Furthermore, leptin injected into the VTA reduces anxiety-like behaviors, and removal of LepR (using AAV-cre technology) from VTA enhanced anxiety-like behaviours (Liu et al., 2015). More specfically, removal of LepR from dopamine neurons (in Dat-Cre mice) also increases anxiety in the elevated plus maze, and is restored by blocking D1 receptors in CeA (Liu et al., 2011). Lastly, leptin is implicated in the modulation of food-related motivation and decision-making via a plastic cortical circuit encompassing the insular cortex. This was obtained through a protocol of self-imposed binge eating-withdrawal cycle in female rats, associated with electrophysiological recordings (Kirson et al., 2022). Taken together, these data support a role for (lack of) leptin in anxiogenesis that engages the dopamine system and decision-making processes.

#### 3.2.3 Ghrelin

Ghrelin is a hormone produced in the stomach, with orexigenic actions exerted through a distributed network that include prominently the hypothalamic arcuate nucleus (through NPY/AgRP neurons). Consistent with effects of AgRP activation to promote a negative valence teaching signal (Betley et al., 2015), ghrelin also conditions an aversion (Schéle et al., 2017), although the precise circuitry through which it elicits this effect has yet to be elucidated. Ghrelin also targets the VTA-nucleus accumbens (NAcc) dopaminergic reward pathway (Jerlhag et al., 2006, 2007) promoting food reward (Egecioglu et al., 2010) and food-motivated behaviour (Skibicka et al., 2012; Skibicka and Dickson, 2011; Méquinion et al., 2013). Patients with anorexia nervosa have high concentrations of plasma ghrelin (Tolle et al., 2003), and this distinguishes them from constitutionally thin women (Germain et al., 2007) and cachectic patients affected with cancer (Blauwhoff-Buskermolen et al., 2017). One possible explanation for the apparent contradiction that they do not eat despite having high ghrelin levels, could be explained ghrelin receptor desensitisation, with ghrelin resistance further contributing to reduced food intake in patients with anorexia nervosa (Garcia-Gil et al., 2022). One of the mechanisms proposed to explain the persistence of food avoidance despite high ghrelin levels is the link between ghrelin and the reward system (and note that ghrelin receptors are present on almost 50% of VTA dopamine neurons (Abizaid et al., 2006)). Thus, hyperghrelinemia could stimulate the dopamine reward system in the presence of starvation-associated cues, and this could lead patients to perceive their control over food intake as rewarding. Studies investigating the relationship between ghrelin and emotional reactivity are still controversial (Méquinion et al., 2013), but notably, in rats, it was found that chronic central ghrelin administration induced an increase in anxiety- and depression-like behaviour (Hansson et al., 2011), an effect that was reduced by both gastrectomy (Salomé et al., 2011) and antisense DNA for ghrelin (Kanehisa et al., 2006). These effects on mood and anxiety have been attributed to an impact of ghrelin on serotonergic transmission in structures such as the amygdala, in which knocking out the ghrelin receptor caused decreased expression of serotonergic receptors (Hansson et al., 2014). However, as reviewed by Fritz et al. (2020), its role in the modulation of anxiety-like behaviors in rodents remains unclear, since it strongly depends on the amount, duration and quality of stress exposure, and ghrelin receptor signalling appear to exert protective effects during chronic stress. Ghrelin also appears to impact decision-making by increasing impulsive choice (Anderberg et al., 2016). Rats treated with intracerebro-ventricular infusions of ghrelin before a delay discounting test displayed a stronger preference for small, immediate rewards over larger but delayed ones (Anderberg et al., 2016). This may have relevance for patients with anorexia nervosa since they appear to have an increased sensitivity for delayed gratification (Steinglass et al., 2012; Steward et al., 2017; Decker et al., 2015). It is now considered as a specific cognitive trait of these patients compared to most psychiatric disorders in which patients exhibit higher delay discounting associated with increased impulsivity (Amlung et al., 2019). Therefore, patients with anorexia nervosa display high plasma levels of ghrelin that do not lead to physiologic ghrelin-induced behaviors such as increased motivation for food intake and increased impulsivity. However, recently published data provided evidence that ghrelin could increase sensitivity to delayed gratification in healthy participants. Indeed, adults exposed to ghrelin infusion exhibited higher sensitivity to delayed gratification in a monetary delay discounting task (Pietrzak et al., 2023). These unexpected results add to previous findings of a positive correlation between plasma levels of desacyl-ghrelin and increased preference for delayed rewards in patients with acute anorexia nervosa (Bernardoni et al., 2020). According to these results it appears that, besides the motivational drive toward food in healthy humans, ghrelin is also involved in other forms of cognitive control, such as the ability to forego immediate gratification in favor of rewards that are distant in time.

#### 3.2.4 Orexin

Another population of interest in the regulation of food intake are the orexins neurons. Located in the lateral hypothalamic area (LHA), these neurons are controlled both by inhibitory tone exerted by the AgRP neurons (Garau et al., 2020) and by ghrelin receptor signaling. Orexins, also known as hypocretins, are neuropeptides with diverse behavioural effects relevant for anorexia nervosa. While the most prominent role of orexin relates to wakefulness, arousal and sleep (Chemelli et al., 1999), orexins also have well studied effects on feeding behaviour (Sakurai et al., 1998), physical activity (Hagan et al., 1999; Kotz et al., 2002), reward, motivation (Harris et al., 2005), stress (Samson et al., 2002) and anxiety (Suzuki et al., 2005). Orexins are produced and released by a discrete population of neurons in the LHA with projection extending to numerous brain areas (Sakurai et al., 1998; de Lecea et al., 1998; Broberger et al., 1998). Upon food restriction orexin neurons respond and release orexins (Sakurai et al., 1998; Fujiki et al., 2001), and in the ABA mouse model a large proportion of these orexin neurons are activated (Schéle et al., 2023), indicating a possible role for the orexin system in anorexia nervosa. There have been a few studies examining circulating orexin levels in patients with anorexia nervosa with very divergent results. In four studies, orexin was found to be increased, decreased and unchanged in patients with anorexia nervosa (Sauchelli et al., 2016; Bronsky et al., 2011; Janas-Kozik et al., 2011; Steward et al., 2016). Furthermore, several studies have shown a link between anxiety and orexins. Narcolepsy is a rare sleeping disorder caused by defective orexin signalling, and the prevalence of anxiety in narcoleptic patients has been reported to be as high as 33–35% (Abenza-Abildua et al., 2023; Fortuyn et al., 2010). Moreover, central orexin injections, including intracerebroventricular injections and focal injections into various brain regions, increase anxiety in a variety of animal behavioural tests (Suzuki et al., 2005; Avolio et al., 2011; Li et al., 2010; Lungwitz et al., 2012). However, anxiety is also increased in orexin neuron and orexin receptor deficient mice (Abbas et al., 2015; Khalil and Fendt, 2017). Orexins are well known to promote locomotor activity and have been proposed to be involved in food seeking behaviours related to locomotion. An interesting hypothesis by Peleg-Raibstein *et al*, suggests that orexin may act as a nutrient-gated switch, involved in the decision process of either consume food or keep seeking once a food item has been found (Peleg-Raibstein et al., 2023). This hypothesis is based on both *in vitro* and *in vivo* experiments showing that orexin neurons are inhibited, following ingestion of calorie dense food such as glucose, while activated following the ingestion of less required nutrients such as non-essential amino acids (Karnani et al., 2011; Viskaitis et al., 2022; Burdakov et al., 2005). Thus, when a calorie dense food is found, loss of orexin signalling leads to the termination of exploratory locomotion and consummatory behaviour start. In contrast, when less valuable food are found and ingested, orexin signalling continues, reprioritizing continued seeking for more essential food.

### 3.3 The role of the reward system and neurocognitive components

As previously described, food related decisions take into account reward expectation, depending on the context and needs. Berridge et al. (2009) define reward with three components: liking (i.e. hedonic value), wanting (i.e. motivation to obtain the reward), and reinforcement learning. Reward expectations related to a choice are encoded within the reward system involving OFC, medial prefrontal cortex (PFC), amygdala, NAcc, VTA (Ikemoto, 2010) and mostly dopamine transmission (Ikemoto, 2010; Höflich et al., 2019).

In anorexia nervosa, the functional structures involved in food decision making are imbalanced, accounting for the mismatch between the actual nutritional state and the decisions concerning food intake. Impairments of the reward circuitry are widely supported (Guillaume et al., 2015; Di Lodovico et al., 2022), postulating: 1) aberrant reward attribution to stimuli that are generally negatively encoded in healthy subjects, such as hunger sensations and excessive physical activity (Steinglass and Foerde, 2018; Keating et al., 2012), and 2) lack of reward associated with food, that results in a decrease of food intake. Davis & Woodside (Davis and Woodside, 2002) claim that the decreased hedonic value of food can be understood in the context of a general tendency to anhedonia. Nevertheless, Klein et al. (2006) reported an increased consumption of non-caloric sweeteners compared to healthy controls, suggesting preserved appetence for sweet taste. Moreover, food seems to be a central concern for patients (Keating et al., 2012). Low or high calorie food could have positive hedonic value for patients, but the incentive value of high calorie food is decreased compared to healthy subjects (Cowdrey et al., 2013). In this respect, food decision in anorexia nervosa is likely driven by altered reward processing in addition to other components such as cognitive control and emotional status. If the reward system allows discrimination between positive or negative valuation of food, cognitive control, mostly exerted by the PFC, allows avoidance of negatively valued food, approach of positively valued food, and adaptation of the behavioural response to the changing environmental needs, hence promoting behavioural flexibility (Gläscher et al., 2012; Miller and Cohen, 2001; Nagano-Saito et al., 2008; Granon and Changeux, 2012). The PFC integrates the ensemble of environmental factors that can interfere with, or influence food intake, hence adjusting food choice in a flexible way. Impaired PFC efficiency, characterizing both anorexia nervosa (Bishop, 2009) and anxiety (Strober, 2004), may contribute to their restrained, cautious, regimented and perfectionistic choices (Strober, 2004), also driven by personality traits such as harm avoidance, and to the obsessive- and compulsive-like manifestations (Steinglass et al., 2010), such as safety behaviours. Furthermore, recent studies have indicated that an overactive medial PFC-striatal circuitry is associated with the development of rigid behaviours and excessive wheel running, which in turn results in weight loss in the ABA model (Santiago et al., 1991; Milton et al., 2021). The authors have postulated that the hyperactivity of the fronto-striatal circuits may result in an excessive control of eating behaviours, which in turn leads to food avoidance. This overcontrol of eating behaviours may subsequently lead to the development of self-restriction and compulsive physical exercise.

Cognitive rigidity may explain the crystallization of food avoidance behaviours through mechanisms such as pathological fear learning. Pathological fear learning involves: 1) preparation to the feared object, 2) automatic recruitment of the fear circuit, and 3) encapsulation of the fear reaction, providing resistance to rationalisation and change (Strober, 2004). Besides the fear of gaining weight, patients also experience a primary fear for food (Brown and Levinson, 2022) by a mechanism of associative learning (Strober, 2004). Finally, cognitive rigidity may also contribute to the diminished efficiency of fear extinction found in anorexia nervosa (Frank, 2021).

### 3.4 How to assess food decision making in animal models?

Still unexploited on anorexia nervosa-like phenotypes, the *Mouse Gambling Task* (MGT) offers a valid and original approach by mimicking uncertainty of outcomes in a complex situation of choice (Pittaras et al., 2016, 2018; Cressant et al., 2013). The MGT is an adaptation of the Iowa Gambling Task developed by Damasio and Bechara (Bechara et al., 1994) for use with the mouse model. The task is conducted within a maze comprising four arms, two of which offer high probabilities of obtaining a small reward (i.e. food pellets) and the other two offer low probabilities of obtaining larger rewards and high probabilities of receiving a penalty (i.e. quinine pellets). Initially, the arms offering larger rewards may be perceived as more advantageous than those offering smaller rewards. However, over time, the animal is more likely to earn a greater amount of reward if it favours smaller rewards with a higher probability of obtaining them. The MGT is able to highlight individual variability in choice, demonstrating the diverse strategies employed by animals.

The MGT is sensitive to alterations of the environment (Pittaras et al., 2022), and unmasks rigid and/or risky behaviours according to individual vulnerability. This test could be useful to challenge decision making alterations on a mouse model mimicking symptoms of anorexia nervosa, and to assess vulnerability to starvation, environmental factors and circuits involved in the pathogenesis of anorexia nervosa.

The delay discounting task (DDT) assesses subjective value calculation in function of the reward's magnitude and delay of receipt (Mitchell, 2014; Richards et al., 1997). Food is normally used as a reward in animals, while humans usually choose among different amounts of money (Mazur, 1987). Conflicting evidence was reported for patients with anorexia nervosa despite the hypothesis of higher tolerance for delayed rewards (Ritschel et al., 2015; Weinert et al., 2022). Food restriction at 80–85% appeared to decrease impulsive choices specifically in female mice. This intriguing result suggests a gender-related difference in sensitivity to food deprivation. Other components of anorexia nervosa, such as physical activity, also affect delay discounting. A single bout of wheel running decreased sensitivity to reinforcement amount and delay (Strickland et al., 2016), affecting the processes that underlie impulsive decision-making in opposing ways.

In a conditioned place preference test (Kucukdereli et al., 2023), mice undergoing repeated exposure to a stressor, and displaying anxiety-prone phenotypes, developed a preference for starvation-like states produced by the optogenetic stimulation of AgRP neurons. This is an example of how this test could be interesting to investigate food decision-making in animals, but to our knowledge, this paradigm is actually unexploited for protocol of food avoidance.

## 4 Therapeutic perspectives

To modify the maladaptive attention patterns to food cues, food-related attention bias modification training (ABMT) seems a promising remediation strategy. ABMT is a procedure that enables patients to shift their attention from unpleasant to pleasant information, and effectively reduced anxious (Liang and Hsu, 2016) and depressive symptoms (Woolridge et al., 2021). In anorexia nervosa, patients are trained to watch high-calorie food images to increase their salience and attractiveness, in order to modify anxiety-related eating behaviours (Mercado et al., 2020). The effectiveness of ABMT could be explained by two mechanisms: (a) the attention control model, which suggests that ABMT increases general attentional control and improves emotional regulation, and (b) the re-evaluation model, which suggests that ABMT changes the subjective value or the threat response of the stimuli by repeated approach or exposure. However, the clinical efficacy of such procedures remains to be determined.

The focus of exposure therapies should be adapted to the specific pattern of abnormal association in each patient (Garcia-Burgos et al., 2023). For instance, in patients presenting aberrant learned associations between food and disgust, it is disgust extinction that should be targeted. To rewire the brain's associations, some promising approaches could be the use of cognitive enhancers, such as d-Cycloserine, during exposure therapy to empower the development of healthy associations. In chronic and resistant anorexia nervosa, habit-centered approaches could better target behavioural responses to food-associated stimuli. *In fine*, the exposure to specific contexts of food consumption (i.e., restaurants) in addition to the stimuli could more extensively counteract anxious responses and behavioural avoidance of food stimuli (Garcia-Burgos et al., 2023).

Another focus could be on interoceptive remediation. Anorexia nervosa has been associated with impaired interoceptive sensitivity and awareness, particularly in the cardiorespiratory domain. Evidence suggests that individuals with anorexia nervosa inaccurately perceive cardiorespiratory sensations, especially during the anticipation of eating (Khalsa et al., 2018). These interoceptive disturbances are often associated with emotion dysregulation, distress intolerance and an inability to discriminate between emotional states and bodily sensations such as hunger. In this context, heart rate variability-based biofeedback has been shown to be effective in improving interoceptive abilities (Meyerholz et al., 2019). This technique allows individuals to receive real-time feedback on their autonomic nervous system activity and learn self-regulation skills, particularly through breathing exercises. Improving interoception could promote better emotional regulation (BecharaAntoine and Damasio, 2005; Wollast et al., 2022; Bechara and Damasio, 2002) and restore the perception of hunger and satiety. Its use has shown promising results in improving cardiac function (Schumann et al., 2019), reducing anxiety (Van Der Zwan et al., 2015), and reducing physical and psychological distress in children and adolescents (Dormal et al., 2021). Thus, biofeedback interventions may help individuals with anorexia nervosa to develop adaptive coping strategies to manage anxiety and distress associated with eating, thereby facilitating meal-targeted interventions. Biofeedback techniques offer a non-invasive and patient-centered approach that can be tailored to the specific needs of individuals with anorexia nervosa. By addressing interoceptive dysfunction, such interventions have the potential to improve patients' food decision-making processes (Meyerholz et al., 2019), support treatment outcomes, and promote long-term recovery.

Recently, neuromodulation techniques, such as deep brain stimulation (DBS), transcranial magnetic stimulation (TMS), transcranial direct current stimulation (tDCS), have been applied in treatment-refractory anorexia nervosa, even though their efficacy needs further confirmation (Shaffer et al., 2023).

DBS is an invasive neuromodulation technique. The procedure involves the implantation of electrodes into deep brain structures, such as the basal ganglia, which are then connected to an implanted pulse generator. The principal objective is to modulate neural activity via the application of extracellular electric fields (McIntyre and Squire, 2009). In recent studies of anorexia nervosa, DBS was found to elicit an altered response in reward-related regions, but not during exposure to food stimuli (Oudijn et al., 2023). Improvements in anxiety and depression were reported at one-year follow-up, while the effects on BMI were contradictory (Lipsman et al., 2017; Scaife et al., 2022). Given the inconsistency of findings, the limited sample sizes, and the lack of comparability between studies, the evidence currently available is insufficient to suggest that DBS may be a viable solution for improving food decision-making in anorexia nervosa. The invasive nature of this device differentiates DBS from other neuromodulation techniques that have demonstrated preliminary efficacy in the treatment of anorexia nervosa, such as TMS and tDCS.

TMS entails the application of a magnetic coil to a targeted region of the scalp, which induces an electric field in the brain, thereby modulating cortical excitability (Rossi et al., 2009). In trials on anorexia nervosa, TMS was primarily targeted on the dorsomedial and dorsolateral PFC. The primary effects of this technique have been observed in the reduction of depressive symptoms, restoration of weight, flexibility, and core symptoms of anorexia nervosa (Gallop et al., 2022). Notably, a reduction in cerebral blood flow in the amygdala following TMS was found to be associated with greater sustained weight gains at 18 months (Dalton et al., 2021). This intriguing finding could suggest that a reduction in fears and anxiety around food could have mediated this improvement, allowing a gain in flexibility of food decision-making.

tDCS is another non-invasive method for modulating cortical excitability. This safe and well-tolerated technique is performed by applying a direct current between 1 and 2 mA between two electrodes of opposed polarity on the scalp at a distance of approximately 35 cm (Been et al., 2007). A recent systematic review on the use of tDCS on patients with anorexia nervosa retrieved five trials, all targeting the dorsolateral PFC, a region that is particularly involved in cognitive control (Chmiel et al., 2023). Preliminary evidence indicated an effect on psychopathological dimensions of anorexia nervosa, BMI and depression. Interestingly, in a non-eating disordered sample, the application of tDCS on the dorsolateral PFC resulted in a significant decrease in risk-taking on a decision-making task (Fecteau et al., 2007). As with DBS, limitations such as insufficient sample sizes, and inconsistent methodologies and results, must be overcome before confirming the efficacy of non-invasive neuromodulation on anorexia nervosa.

## 5 Conclusion

Food decision-making in anorexia nervosa seems driven by different factors from those regulating approach and reward in healthy individuals. Cognitive, emotional, neurobiological and neuroendocrine dysregulations may, at least partially, account for the transformation of motivated approach into anxiety-driven fearful avoidance of specific food categories. The ABA mouse model allowed us to explore the bidirectional relationship between anxiety and anorexia-like behaviours. To progress further, animal models of decision making under conditions of uncertainty, such as the MGT or the delay discounting task could be exploited to test the role of anxiety components on food choice and avoidance. Therapeutic strategies aimed at dissociating food stimuli from vigilance and fear response, or to improve emotional regulation, could be useful in restoring a healthy decisional process in anorexia nervosa.

## Funding sources

This work was supported by University of Paris (LDL, HH, CTM, VT, OV), University of Lille (LDZ, OV) and Institut National de la Santé et de la Médicale (LDL, HH, CTM, VT, OV). LDL is the recipient of PhD fellowship from the “Institut National de la Santé et de la Médicale”. CTM is the recipient of a PhD fellowship (N◦ FDM202006011161) funded by “Fondation pour la Recherche Médicale”. HH is supported by Agence Nationale pour la Recherche (ANR Rewan-19-CE37-0020-01). RA is supported by ERANET-NEURON 2018, grant number MIGBAN FKZ: 01EW1906A, the Swedish Research Council for Medicine and Health (2018–02588), Vrienden van het UMC and the Netherlands organisation for Scientific Research (ALWOP.137, OCENW.M.22.111, Gravitation grant 024.004.012). SLD is supported by the Swedish Research Council (2022-00713), NovoNordisk Fonden (NNF0078215), Hjärnfonden (FO2023-0437) and Swedish Government and the county councils in the ALF agreement (ALFGBG-965364).

The EBRA project has received funding from the European Union's Horizon 2020 research and innovation program under grant agreement No 825348.

## Declaration of generative AI in scientific writing

The authors declare that they have no referred to the writing process and have not used AI to analyze and draw insights from data as part of the research process.

**Fig. 1:**
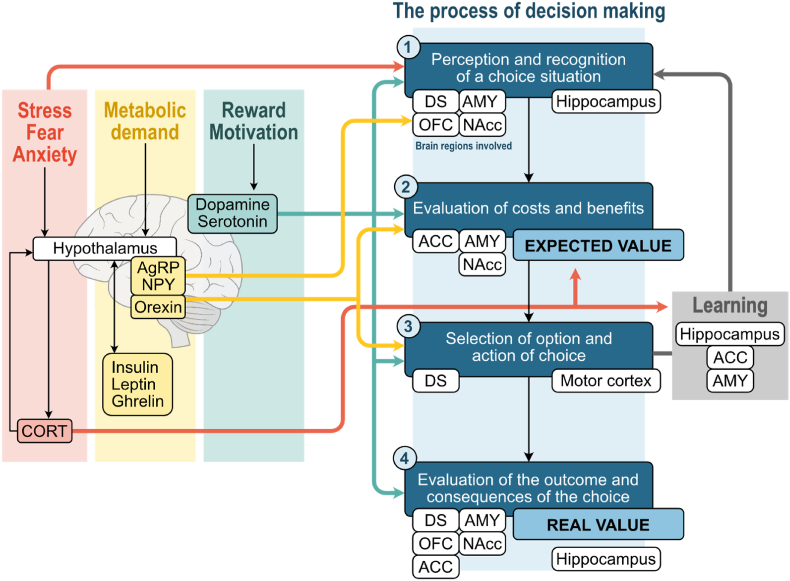
Fig. 1. Food-related decision making associated neural structures and hormonal factors likely influencing the different components of food decision-making. (Based on Doya, 2008) ACC, anterior cingulate cortex; AgRP: Agouti related protein AMY, amygdala; CORT, corticosteroïdes; DS, dorsal striatum; NAcc, nucleus accumbens; OFC, orbitofrontal cortex; NPY, Neuropeptide Y.

## Declaration of competing interest

The authors declare that they have no known competing financial interests or personal relationships that could have appeared to influence the work reported in this paper.
